# Automated Road Marking Wear Assessment via Multimodal Fusion of LiDAR Intensity and YOLOv11 Semantic Segmentation

**DOI:** 10.3390/s26175549

**Published:** 2026-08-31

**Authors:** Pin-Yung Chen, Shu-Wei Hsu, Po-Wei Chen, Hao-Chu Lin, Chien-Chiang Tung, Shin-Hung Chang

**Affiliations:** 1 Department of Vehicle Engineering, National Pingtung University of Science and Technology, Pingtung 912301, Taiwan; a0931562862@gmail.com (S.-W.H.); s993109@gmail.com (P.-W.C.); 2 Department of Mechanical and Computer-Aided Engineering, Overseas Chinese University, Taichung 407105, Taiwan; hclin@ocu.edu.tw; 3 Graduate Institute of Applied Science and Technology, National Taiwan University of Science and Technology, Taipei 106335, Taiwan; jiannchyang@gmail.com; 4 Department of Electrical Engineering, Chung Yuan Christian University, Taoyuan 320314, Taiwan; shc@cycu.edu.tw

**Keywords:** road marking inspection, LiDAR intensity, semantic segmentation, sensor fusion, YOLOv11, mobile mapping, intelligent transportation

## Abstract

**Highlights:**

**What are the main findings?**

- A mobile multimodal road inspection system was developed by integrating LiDAR intensity, camera-based semantic segmentation, GNSS, IMU, and ROS-based processing.
- A grid-based wear score was constructed by combining reflectance ratio, point density ratio, fusion retention ratio, and geometric coverage.

**What are the implications of the main findings?**

- The proposed workflow converts raw sensing data into grid-based road marking wear scores, review flags, CSV reports, and georeferenced maintenance maps.
- The method provides a practical sensing framework for intelligent transportation inspection and road asset maintenance management.

**Abstract:**

Road markings support lane guidance, traffic regulation, and machine perception, but their field inspection still depends largely on manual surveys or local retroreflectivity measurements. This study presents a vehicle-mounted inspection framework that fuses LiDAR intensity with camera-based semantic segmentation for automated road marking wear assessment. The platform integrates LiDAR, a stereo camera, IMU, GNSS, and an industrial computer. LiDAR-inertial mapping provides spatial alignment, while ground filtering, region-of-interest extraction, and adaptive intensity thresholding generate preliminary marking candidates. A YOLOv11 segmentation model produces pixel-level marking masks, and LiDAR candidates are projected onto the image plane for semantic confirmation. Confirmed points are accumulated into grid cells and evaluated using reflectance, point density, fusion retention, and geometric coverage indicators. In a representative route, 2441 grid units were analyzed: 1210 units were valid for formal grading, with 1060 good, 131 slightly worn, and 19 moderately worn units; 1231 units were reserved for review. The mean composite score of the valid grids was 0.906. A five-report aggregate further showed that 87.2% of the units received valid wear categories. The results indicate that multimodal fusion transforms road marking inspection into a quantitative, spatially referenced, and reportable process.

## 1. Introduction

Road markings, including lane lines, directional arrows, zebra crossings, and pavement symbols, are essential traffic control components that provide visual guidance for human drivers and reliable environmental cues for advanced driver assistance systems (ADAS) and autonomous vehicles. As road markings are continuously exposed to traffic loading, ultraviolet radiation, rainfall, and pavement aging, their visibility and retroreflective performance gradually deteriorate, reducing driving safety and increasing maintenance requirements. Consequently, regular inspection and condition assessment have become indispensable components of road asset management. Conventional inspection methods mainly rely on manual visual surveys or portable retroreflectometers, which are labor-intensive, time-consuming, and difficult to apply efficiently over large-scale road networks [1,2,3,4].

Recent advances in mobile mapping systems have enabled automated road inspection using vehicle-mounted LiDAR and vision sensors. LiDAR provides accurate three-dimensional geometric information, together with reflectance intensity, making it suitable for road surface extraction, localization, and preliminary road marking detection [5,6,7,8,9]. Camera-based semantic segmentation provides dense visual information and pixel-level classification for identifying different road marking types under various traffic scenarios [10,11,12].

Nevertheless, each sensing modality has inherent limitations. LiDAR intensity is affected by sensing distance, incidence angle, pavement material, and highly reflective non-marking objects, whereas image-based approaches remain sensitive to illumination variation, shadows, glare, motion blur, and severely deteriorated markings. Consequently, a single sensing modality often produces unstable inspection performance in complex road environments.

To improve perception robustness, multimodal sensor fusion has become an effective strategy for integrating complementary sensing information. Accurate LiDAR–camera calibration enables three-dimensional point clouds to be projected onto image coordinates, allowing geometric observations to be verified using semantic information extracted from images [13,14,15]. Such fusion techniques have been extensively investigated in intelligent transportation systems and autonomous driving, particularly for object detection, localization, and environmental perception. However, relatively few studies have focused on maintenance-oriented road marking assessment, especially those combining LiDAR intensity, semantic segmentation, and quantitative wear evaluation within a unified inspection framework.

To address these limitations, this study proposes a vehicle-mounted multimodal road marking inspection framework integrating LiDAR intensity analysis, camera-based semantic segmentation, LiDAR–camera fusion, and grid-based wear assessment. LiDAR–inertial mapping provides spatially referenced point clouds, while semantic segmentation verifies candidate road marking regions through projection-based fusion. The fused observations are subsequently converted into quantitative wear scores and georeferenced maintenance reports using a grid-based evaluation strategy. The proposed framework provides an integrated and automated workflow for converting multimodal sensing data into spatially referenced road marking assessment outputs that may support road inspection and maintenance planning.

## 2. Materials and Methods

### 2.1. System Architecture and Vehicle-Mounted Platform

The vehicle-mounted platform consisted of an Ouster OS1-32 Ultra LiDAR (Ouster, Inc., San Francisco, CA, USA), a ZED stereo camera (Stereolabs, San Francisco, CA, USA), an Xsens MTi-G-710 IMU/GNSS unit (Xsens Technologies B.V., Enschede, The Netherlands), and a Nuvo-10108GC industrial computer (Neousys Technology Inc., New Taipei City, Taiwan). The industrial computer was equipped with an Intel Core i9-13900E processor, 32 GB of DDR5 memory, and an NVIDIA GeForce RTX 4080 SUPER GPU with 16 GB of memory. The sensing and computing devices were integrated on a passenger vehicle, and ROS was used for sensor data acquisition, communication, and processing.

The Ouster OS1-32 Ultra provided a maximum measurement range of 200 m, a measurement accuracy of ±3 cm, a 360° horizontal field of view, and a vertical field of view from −22.5° to +22.5°. It supported horizontal resolutions of 512, 1024, and 2048 points per revolution and selectable rotation frequencies of 10 or 20 Hz. The ZED stereo camera provided a maximum horizontal field of view of 90°, a vertical field of view of 60°, a nominal depth range of 0.5–20 m, and a maximum frame rate of 100 frames per second.

**Figure 1:**
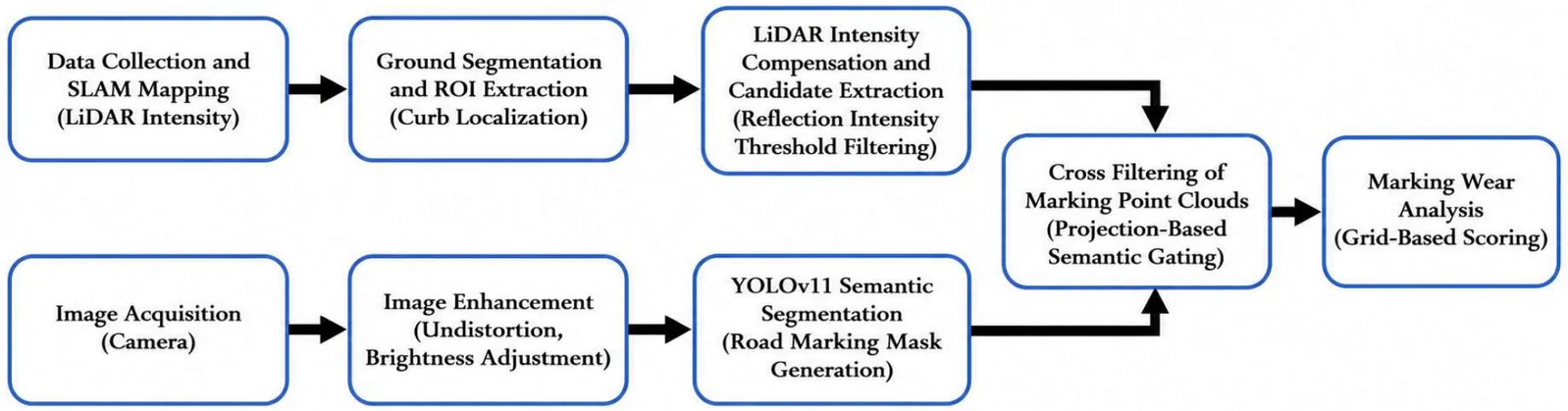
Overall workflow of proposed vehicle-mounted multimodal road marking inspection framework.

The processing pipeline included LiDAR mapping, semantic segmentation, sensor fusion, wear evaluation, and report generation, as illustrated in Figure 1.

### 2.2. LiDAR–Inertial Mapping, Coordinate Alignment, and Calibration

LiDAR–inertial odometry was adopted to provide vehicle pose estimation and construct a consistent point cloud map. GNSS measurements were used to attach approximate geographic references to inspection results. Before sensor fusion, all sensing data were transformed into a unified coordinate system. Intrinsic camera parameters were obtained through checkerboard calibration, while extrinsic LiDAR–camera parameters were estimated offline using a shared calibration target. The resulting rigid transformation was applied during the online projection of the LiDAR points onto the image plane.

### 2.3. Road Surface Candidate Extraction from LiDAR

The first LiDAR processing stage extracts road surface points from the raw point cloud. A slope-based ground filter and statistical outlier removal are applied to eliminate non-road points and isolated noise. The analysis is then restricted to a road-oriented region of interest (ROI) to suppress roadside objects and opposite-lane interference. To improve robustness on curved roads, the ROI is adaptively adjusted using the smoothed IMU yaw rate.

Road markings generally exhibit higher LiDAR reflectance than asphalt; however, the measured intensity is affected by sensing distance, incidence angle, and pavement characteristics. Therefore, range and incidence-angle compensation are applied before adaptive statistical thresholding. The range-compensated intensity is calculated by Equation (1), and the incidence-angle correction is described by Equation (2). An adaptive threshold is then determined from the local intensity distribution using Equation (3), and points exceeding the threshold are retained as preliminary road marking candidates.

(1) $$I^{\prime }=I\times r^{p},\;r=\sqrt{x^{2}+y^{2}+z^{2}}$$

(2) $$I^{\prime }=\frac{I_{r}}{\max\left(\cos\theta ,\epsilon \right)},\;\cos\theta \approx \frac{\left|z\right|}{r}$$

(3) $$\mathrm{thr}=\max\;((\mathrm{median}(I^{\prime })+k_{\sigma }g\mathrm{stdev}(I^{\prime }))s_{\mathrm{thr}},\;I_{\min})$$

In Equation (1), $I_{i}$ is the raw intensity of the i-th LiDAR point, $r_{i}$ is the three-dimensional distance from the LiDAR origin to the point, and $x_{i}$, $y_{i}$, and $z_{i}$ are its coordinates in the LiDAR frame. The parameter p is the range compensation exponent, and $I_{r,i}$ is the range-compensated intensity. Equation (2) further compensates for the incidence angle effect, where $I_{c,i}$ denotes the corrected intensity, $\theta_{i}$ is the approximate incidence angle, and ε is a small positive constant used to prevent division by a near-zero value. Equation (3) calculates the adaptive threshold thr from the median and standard deviation of the corrected intensities. Here, $k_{\sigma }$ is the standard deviation gain, $s_{thr}$ is the threshold scaling factor, and $I_{min}$ is the minimum safeguard threshold. Points with corrected intensity values greater than thr are retained as preliminary road marking candidates.

**Figure 2:**
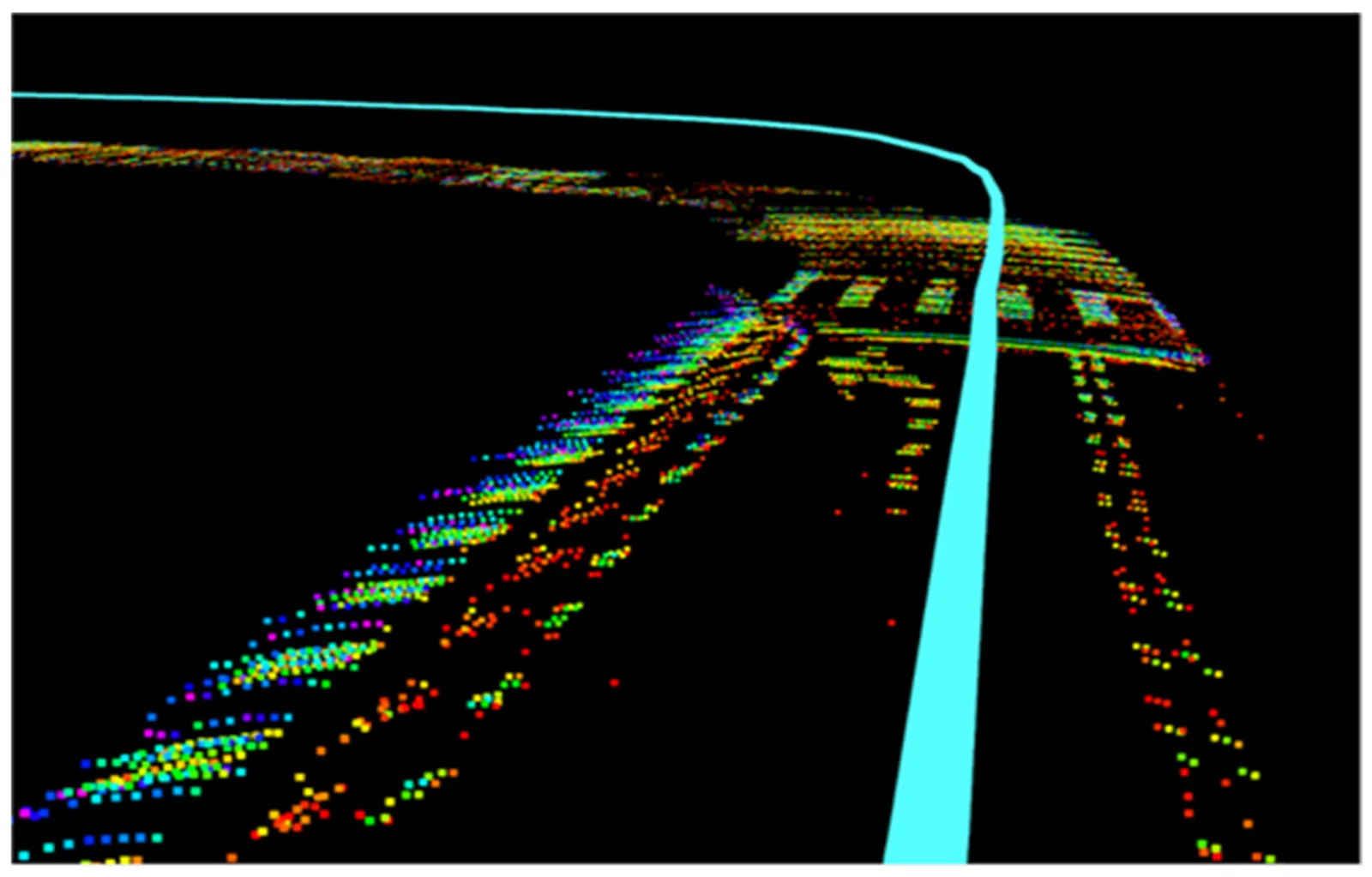
LiDAR-based road marking candidate extraction after ground filtering, ROI extraction, and adaptive intensity thresholding. The point colors are used only for visualization of the LiDAR intensity distribution and do not represent different road marking classes.

As shown in Figure 2, the road marking candidates are extracted after ground segmentation, ROI filtering, and adaptive intensity thresholding. Although intensity filtering effectively highlights road markings, high-reflectance non-marking objects may still be retained, motivating the subsequent semantic verification stage.

### 2.4. Image-Based Semantic Segmentation and Mask Stabilization

The camera branch provides semantic information that complements the geometric and reflectance information obtained from LiDAR. Before segmentation, input images are enhanced using gamma correction, Gray-world white balance, and contrast-limited adaptive histogram equalization to improve robustness under varying illumination conditions.

**Figure 3:**
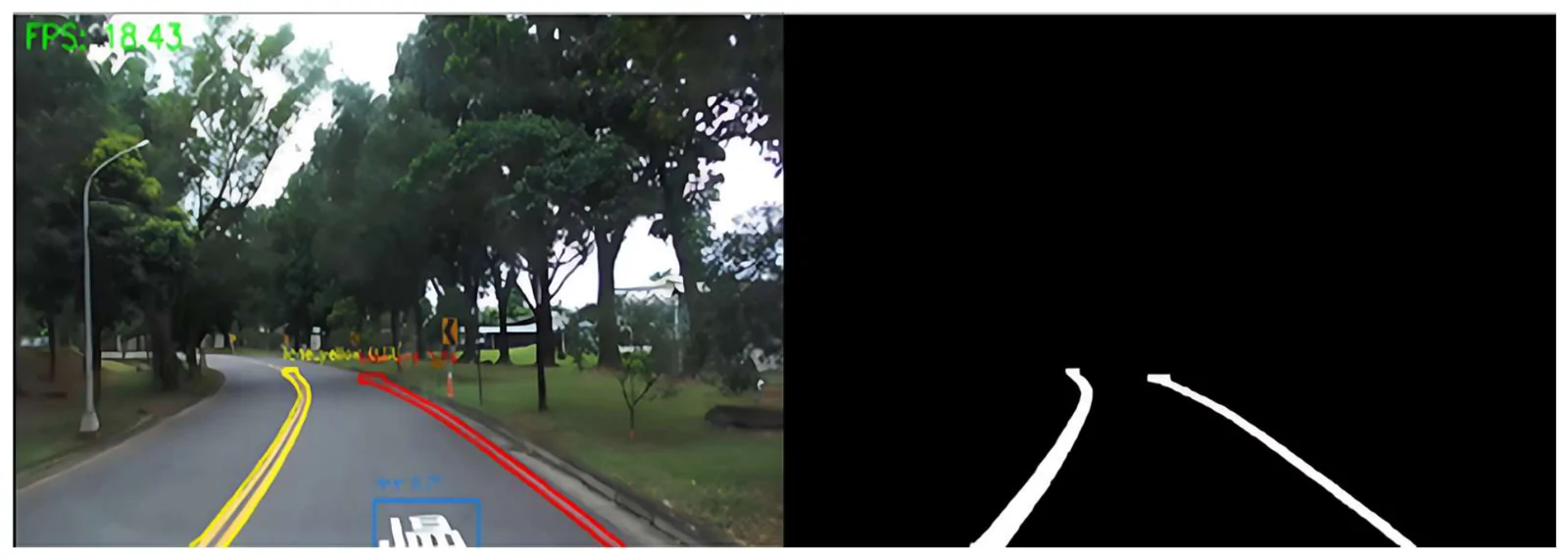
YOLOv11 semantic segmentation results and corresponding binary masks for road markings.

A YOLOv11 segmentation model is adopted to identify lane lines, arrows, and pavement symbols, producing pixel-level binary masks for subsequent sensor fusion. To improve segmentation stability, lightweight post-processing, including morphological dilation and short-term mask stabilization, is applied to suppress fragmented detections and temporary recognition failures. Frames that do not satisfy the minimum mask validity requirement are excluded from fusion to prevent unstable image results from removing valid LiDAR candidates. Figure 3 illustrates an example of the semantic segmentation results and the corresponding binary masks. YOLOv11 was selected because it provides pixel-level segmentation masks within a unified and deployment-oriented framework suitable for integration with the vehicle-mounted processing system. Its available model variants also allow a practical trade-off among validation performance, inference time, model size, and computational demand.

To select an appropriate model scale, the YOLOv11 n, s, m, l, and x configurations were compared in terms of model complexity, inference efficiency, and validation performance using the reference characteristics reported by Ultralytics [11]. The lightweight configurations provide faster inference and have lower computational requirements, whereas the larger configurations provide stronger validation performance at increased computational cost. Considering that the vehicle-mounted platform was equipped with a high-performance industrial computer and that segmentation reliability was important for subsequent LiDAR–camera fusion, the x-scale configuration was selected. Accordingly, YOLOv11x-seg was used as the segmentation model in this study and was trained for up to 500 epochs.

### 2.5. Projection-Based LiDAR–Camera Fusion

The fusion stage combines LiDAR marking candidates with image-confirmed marking regions. Each LiDAR candidate point is transformed from the LiDAR coordinate system into the camera coordinate system using the calibrated extrinsic parameters and projected onto the image plane through a pinhole camera model, as described by Equation (4).

(4) $$u=f_{x}^{\prime }\frac{X}{Z}+c_{x}^{\prime },\;v=f_{y}^{\prime }\frac{Y}{Z}+c_{y}^{\prime }$$

In Equation (4), $X_{C}$, $Y_{C}$, and $Z_{C}$ are the coordinates of a LiDAR candidate point after transformation into the camera coordinate system. The parameters $f_{x}$ and $f_{y}$ denote the focal lengths adjusted to the inference-image resolution, while $c_{x}$ and $c_{y}$ denote the corresponding principal point coordinates. The variables $u_{i}$ and $v_{i}$ are the horizontal and vertical pixel coordinates obtained using the pinhole projection model. Points with $Z_{C}\leq 0$ or with projected coordinates outside the image boundaries are discarded.

Projected points located outside the valid image region or behind the camera are discarded. The binary semantic mask is then used to verify the projected points, and only LiDAR candidates located within valid marking regions are retained as fused marking points, as expressed in Equation (5).

(5) $$P_{\mathrm{fused}}=\left\{p_{i}\in P_{\mathrm{cand}}\;|\;M\left(u_{i},v_{i}\right)=1\right\}$$

Equation (5) defines the semantic gating operation. Here, $P_{cand}$ denotes the set of preliminary LiDAR road marking candidates, $p_{i}$ denotes the i-th LiDAR candidate point, and $\left(u_{i},v_{i}\right)$ denotes its projected pixel coordinates. $M(u_{i},v_{i})$ is the binary road marking mask value at the corresponding pixel. A candidate point is included in the fused set $P_{fused}$ only when $M(u_{i},v_{i})=1$. Thus, the fusion stage retains the LiDAR points supported by the image-based road marking mask and removes candidates outside the valid marking regions.

**Figure 4:**
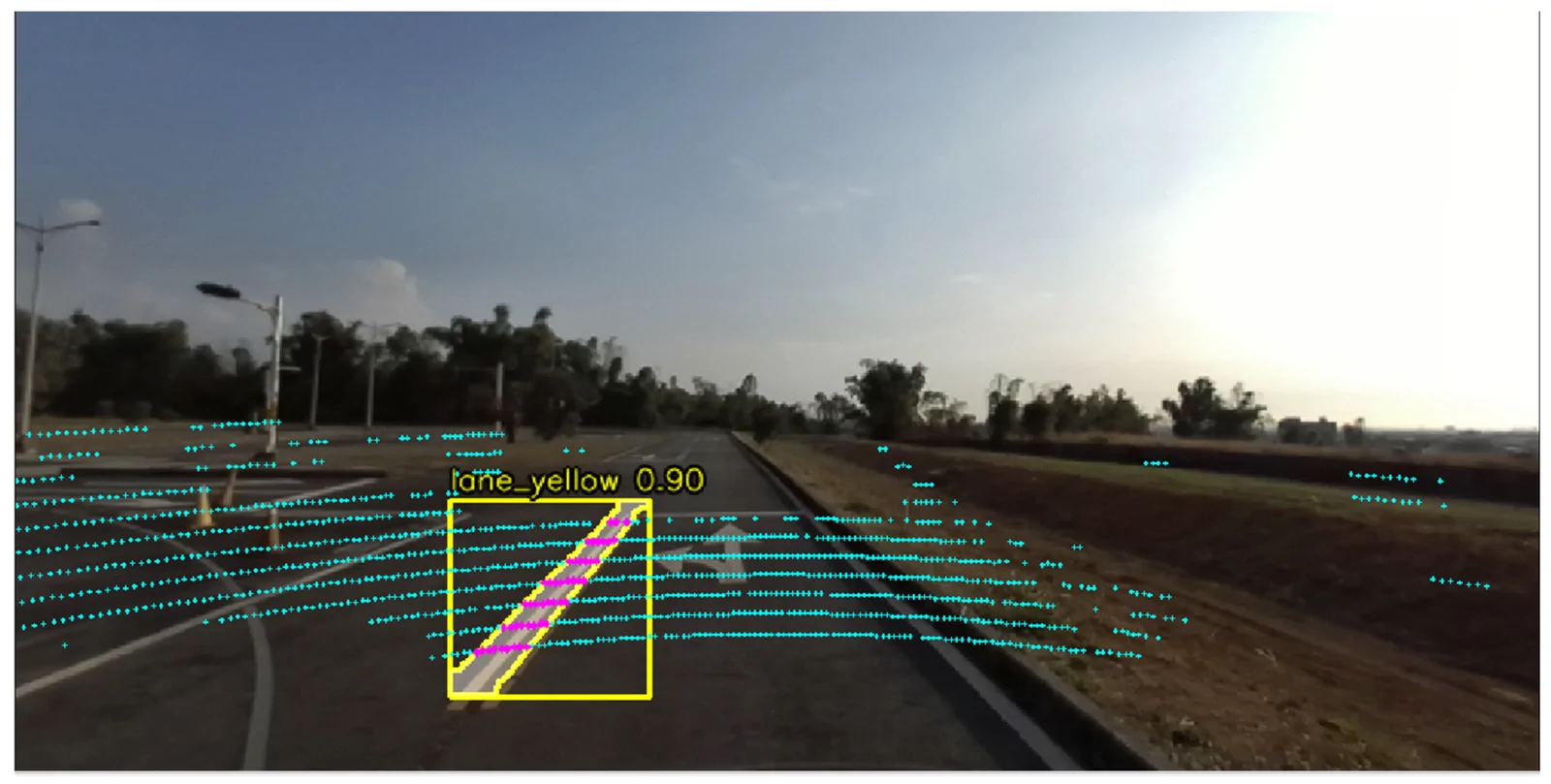
Projection-based LiDAR–camera fusion using semantic mask verification. Cyan points represent the projected LiDAR points, the yellow box and label indicate the YOLOv11 detection result and confidence score, and the magenta region represents the road marking semantic mask.

This projection-based semantic-gating procedure removes high-intensity LiDAR candidates that are not supported by the image-based road marking masks while retaining candidates that are geometrically and semantically consistent with the projected marking regions. Figure 4 illustrates the projection process and semantic mask verification. The resulting fused point set is used for subsequent grid-based wear assessment.

### 2.6. Grid-Based Wear Scoring and Output Generation

After fusion, road marking points are aggregated into fixed spatial grid cells, which serve as the minimum analysis units for wear evaluation. Four quantitative indicators are calculated for each grid cell, including the reflectance intensity ratio, point density ratio, fusion retention ratio, and geometric coverage ratio. These indicators describe the reflectance quality, spatial density, semantic consistency, and continuity of road markings.

The final wear score is computed using the weighted formulation shown in Equation (6), where the four indicators are combined according to predefined weighting factors.

(6) $$S=\frac{W_{I}R_{I}+W_{D}R_{D}+W_{F}R_{F}+W_{G}R_{G}}{W_{I}+W_{D}+W_{F}+W_{G}}$$

Equation (6) calculates the composite wear score S is a normalized weighted combination of four indicators. $R_{I}$ is the reflectance intensity ratio, $R_{D}$ is the point density ratio, $R_{F}$ is the fusion retention ratio, and $R_{G}$ is the geometric coverage ratio. $W_{I}$, $W_{D}$, $W_{F}$, and $W_{G}$ are their corresponding weights. The denominator normalizes the result by the sum of the weights. For the parameter values listed in Table 1, the four weights sum to one. A higher S value indicates stronger reflectance, denser point support, greater semantic consistency, and more complete geometric coverage within the grid.

The scoring weights were empirically assigned according to the relative roles of the four indicators. Reflectance intensity and fusion retention were given higher weights because they represent marking reflectivity and semantic consistency, respectively. Point density was assigned a lower weight because it is affected by sensing range and LiDAR sampling characteristics, while geometric coverage was used as a supplementary continuity indicator. The grading thresholds were empirically defined for the current sensor configuration and may require recalibration for other sensing platforms and road environments.

## 3. Results

### 3.1. Data Acquisition, Experimental Conditions, and Qualitative Results

The experimental data were acquired using the vehicle-mounted platform described in Section 2.1. LiDAR point clouds, camera images, IMU measurements, and GNSS information were recorded during vehicle operation.

The representative field survey covered an approximately 0.52 km road section containing both straight and curved road segments. The system generated 2441 spatial analysis grids along the surveyed section, of which 2214 contained valid observation records. The generated inspection report included grid-level information such as observation scan count, cumulative observation time, cumulative observation distance, and average vehicle speed. Based on the recorded observations, the mean scan-weighted vehicle speed was approximately 18.25 km/h. These speed records describe the actual field survey rather than controlled experiments conducted at predefined vehicle speeds. Meteorological conditions and pavement wetness were not independently recorded and were therefore not quantitatively evaluated in the current study.

Figure 2, Figure 3 and Figure 4 present the outputs of the LiDAR preprocessing, image-based semantic segmentation, and projection-based fusion modules. LiDAR candidates supported by the semantic masks were retained for subsequent grid-based wear analysis.

### 3.2. Road Marking Segmentation Model Selection and Results

Table 2 summarizes the YOLOv11 n, s, m, l, and x model-scale configurations used as the architecture selection reference in this study. The comparison illustrates the trade-off between validation performance and computational efficiency. The lightweight configurations have shorter inference time, smaller parameter count, and lower computational demand, whereas the larger configurations achieve higher validation performance. Among the compared configurations, YOLOv11x achieved the highest reference mAP50–95 of 54.7 but also required the largest numbers of parameters and FLOPs and the longest CPU ONNX inference time. Because the vehicle-mounted platform was equipped with an NVIDIA GeForce RTX 4080 SUPER GPU and reliable semantic masks were important for subsequent LiDAR–camera fusion, the x-scale configuration was selected, and YOLOv11x-seg was used for the segmentation experiments.

**Figure 5:**
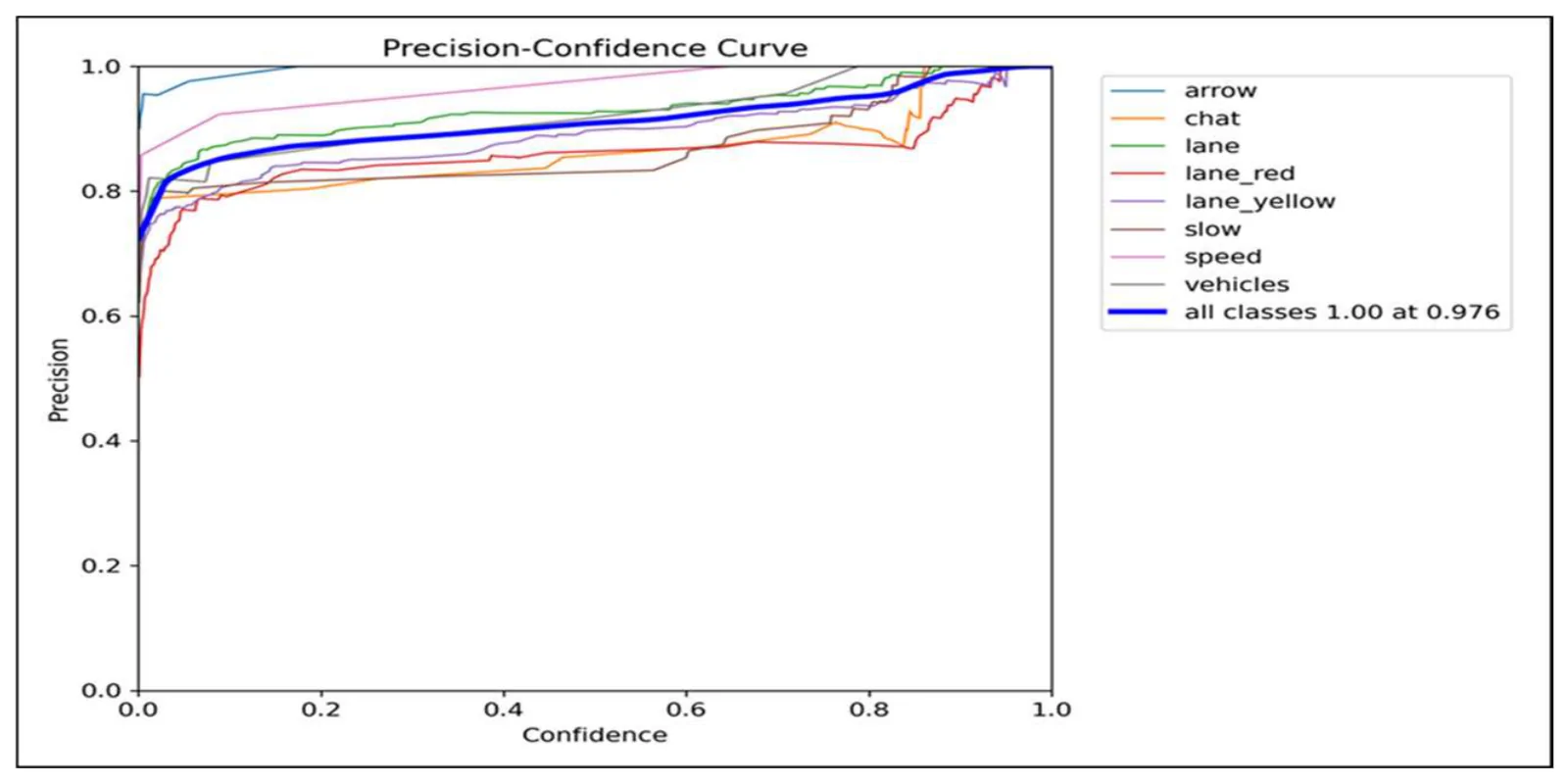
Precision–confidence curves of trained YOLOv11x-seg model for different road marking classes.

Figure 5 shows the class-wise precision–confidence curves. Overall precision increased with the confidence threshold and reached 1.00 at 0.976. The results indicate class-dependent performance; therefore, the segmentation masks were used mainly for semantic verification of LiDAR candidates rather than as the sole basis for wear assessment.

### 3.3. Representative Route Assessment

A representative route was analyzed to evaluate the proposed wear assessment framework. The system generated 2441 analysis grid units, of which 1210 met the grading criteria, and 1231 were assigned to the review category because of insufficient observations or fusion evidence. The review category is intended to prevent unreliable data from being directly classified into wear levels.

**Figure 6:**
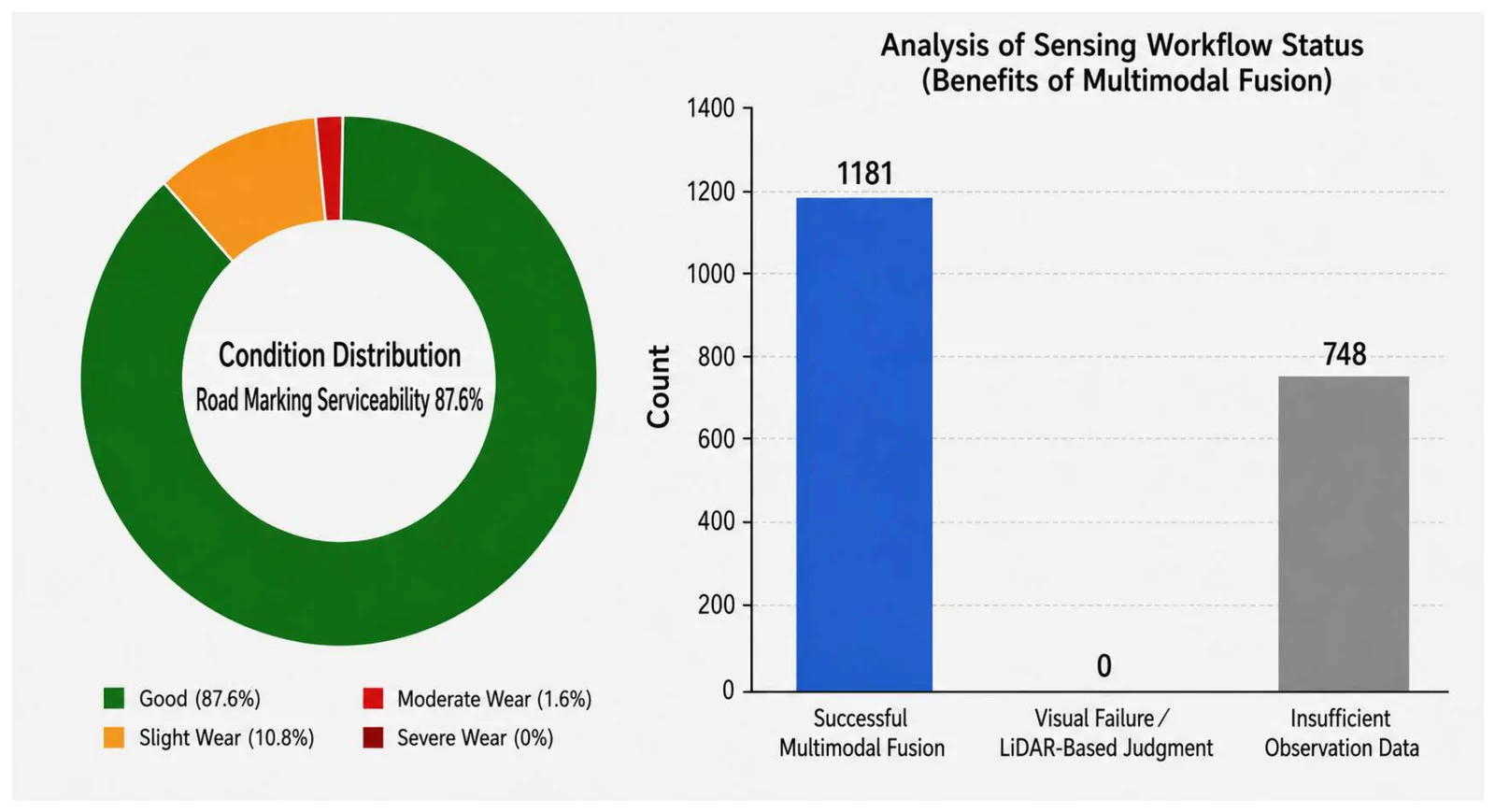
Road marking wear assessment results for representative route.

Table 3 summarizes the grading results. Among the valid grids, 1060 (87.6%) were classified as good, 131 (10.8%) as slight wear, and 19 (1.6%) as moderate wear, with no grids assigned to the severe wear category. The mean composite wear score of the valid grids was 0.906. This value represents the score distribution generated by the proposed framework and should not be interpreted as independently verified classification accuracy. Figure 6 further illustrates the road marking wear distribution and the sensing workflow status for the representative route.

The reported percentages describe the distribution of wear categories generated by the proposed framework rather than independently verified system accuracy. No manual ground-truth labels, expert condition assessments, or professional retroreflectivity measurements were available for the current dataset. Therefore, the agreement between the generated wear categories and the actual road marking conditions could not be quantitatively evaluated in this study. Independent validation using reference measurements and standard classification metrics will be conducted in future work.

### 3.4. Multi-Report Aggregate Assessment

To examine the consistency of the output generation process, results from five inspection reports were aggregated. A total of 4212 grid units were analyzed, with a mean composite wear score of 0.711. Among all units, 87.2% received system-generated wear categories, whereas 12.8% were assigned to the review category because of insufficient observations or fusion evidence. Table 4 summarizes the aggregate grading statistics. These results show that the proposed framework consistently generated composite scores, wear category outputs, and review flags across the five inspection reports while conservatively handling observations with insufficient evidence. However, the agreement between the generated categories and the actual road marking conditions requires independent ground-truth validation.

## 4. Discussion

### 4.1. Advantages of Multimodal Wear Assessment

The proposed framework combines LiDAR intensity with image-based semantic segmentation to provide complementary geometric, reflectance, and semantic information for automated road marking assessment. LiDAR provides three-dimensional location and reflectance information, whereas semantic segmentation supplies the shape and semantic context of road markings. By projecting LiDAR candidates onto the semantic masks, the proposed fusion strategy is designed to remove high-reflectance candidates that are not supported by image-based road marking regions while retaining geometrically and semantically consistent points for subsequent analysis.

The proposed grid-based evaluation converts the multimodal sensing results into structured maintenance-oriented information. Each grid cell contains a composite wear score, a generated wear category, and supporting observation information, allowing potentially abnormal locations to be identified and geographically referenced. In addition, the review mechanism prevents grid cells with insufficient observations or fusion evidence from being directly assigned to formal wear categories. This conservative strategy reduces the risk of assigning unsupported wear grades to dashed lane markings, intersections, curved roads, and partially occluded regions. Nevertheless, the actual reduction in classification errors and the accuracy of the generated wear categories require validation against independent reference data.

### 4.2. Limitations

Several limitations remain in the current study. First, the scoring weights and grading thresholds were empirically predefined for the current sensing configuration and may require recalibration when different LiDAR sensors, pavement materials, road marking types, installation positions, or operating environments are used. A systematic parameter sensitivity analysis was not conducted in the present study.

Second, no independent reference assessment was available to verify the final road marking wear categories. The current dataset did not include manually labeled ground-truth grids, expert visual assessments, or professional retroreflectivity measurements. Therefore, the reported composite scores and category distributions should be interpreted as outputs generated by the proposed framework rather than measures of classification accuracy.

Third, LiDAR–camera fusion remains sensitive to extrinsic calibration errors, synchronization inaccuracies, illumination changes, adverse weather, vehicle motion, and temporary occlusions. These factors may affect point-to-image projection accuracy, semantic mask stability, and the number of valid observations retained for each grid cell. Although grid-level vehicle speed information was recorded in the generated inspection reports, controlled experiments under predefined vehicle speed conditions were not conducted. In addition, detailed operational sensor settings, meteorological conditions, and pavement wetness information were not independently recorded for the current field dataset.

Previous studies have reported that fog and rain can attenuate and scatter automotive LiDAR signals, reduce effective detection range, and alter the intensity and point return distributions [16,17,18]. Background filtering is also important for suppressing spurious roadside LiDAR observations [19]. These effects may further degrade camera visibility and semantic mask stability, thereby reducing fusion retention and increasing the proportion of grid cells assigned to the review category. Because the current dataset did not include controlled adverse weather conditions, these effects were not quantitatively evaluated in this study.

Finally, the geographic outputs generated in this study provide maintenance-oriented location references rather than survey-grade positioning. Future work will focus on systematic parameter optimization, controlled vehicle speed testing, improved sensor synchronization, validation using manually labeled reference grids and expert assessments, comparison with professional retroreflectivity measurements, and evaluation under diverse road and weather conditions.

## 5. Conclusions

This study proposed a vehicle-mounted multimodal road marking inspection framework that integrates LiDAR intensity with camera-based semantic segmentation for automated wear assessment. By combining LiDAR–inertial mapping, road surface candidate extraction, projection-based LiDAR–camera fusion, and grid-based evaluation, the framework converts raw multimodal sensing data into spatially referenced road marking observations and structured inspection outputs.

The experimental implementation showed that the proposed framework can generate semantically verified road marking point sets, grid-based composite scores, wear category outputs, review flags, CSV reports, and georeferenced maintenance maps. The review mechanism also prevents observations with insufficient evidence from being directly assigned to formal wear categories. These outputs provide structured and spatially referenced information that may support road inspection, maintenance prioritization, and road asset management.

However, the accuracy of the final wear categories was not independently validated in the current study. The reported composite scores and category distributions should therefore be interpreted as system-generated assessment outputs rather than verified classification accuracy. Future work will validate the proposed scoring framework using manually labeled ground-truth grids, expert visual assessments, and professional retroreflectivity measurements. Additional work will include parameter sensitivity analysis, controlled vehicle speed experiments, improved sensor synchronization, and evaluation under diverse pavement, illumination, and adverse weather conditions.

## Figures and Tables

**Table 1:** Scoring parameters, grading thresholds, and empirical selection basis used in current implementation.

Parameter	Value	Meaning	Selection Basis
W_I	0.40	Weight of reflectance intensity ratio	Highest weight because intensity directly reflects road marking reflectance quality.
W_D	0.10	Weight of point density ratio	Lower weight because point density is affected by range, vehicle motion, and LiDAR sampling.
W_F	0.35	Weight of fusion retention ratio	High weight because it represents semantic consistency between LiDAR candidates and image masks
W_G	0.15	Weight of geometric coverage indicator	Supplementary indicator of spatial continuity and marking coverage
Good	S ≥ 0.80	Marking is considered to have sufficient quality	Empirically defined for grids with consistently high indicator values
Slight wear	0.65 ≤ S < 0.80	Early degradation requiring attention or tracking	Empirically defined for early quality reduction requiring monitoring
Moderate wear	0.45 ≤ S < 0.65	Noticeable degradation	Empirically defined for clear reductions in multiple indicators
Severe wear	S < 0.45	Severe degradation when sufficient evidence is available	Empirically defined for low composite scores with sufficient observations

**Table 2:** Comparison of YOLOv11 model-scale configurations used as architecture selection reference in this study.

Model	mAP^Val^ 50–95	Speed CPU ONNX (ms)	Parameters (M)	FLOPs (B)
YOLO11n	39.5	56.1 ± 0.8	2.6	6.5
YOLO11s	47.0	90.0 ± 1.2	9.4	21.5
YOLO11m	51.5	183.2 ± 2.0	20.1	68.0
YOLO11L	53.4	238.6 ± 1.4	25.3	86.9
YOLO11x	54.7	462.8 ± 6.7	56.9	194.9

**Table 3:** Representative route’s wear assessment results.

Category	Number of Grid Units	Percentage of All Grids	Percentage of Valid Graded Grids
Good	1060	43.4%	87.6%
Slight wear	131	5.4%	10.8%
Moderate wear	19	0.8%	1.6%
Severe wear	0	0.0%	0.0%
To be reviewed	1231	50.4%	Not included in valid grading
Total	2441	100.0%	1210 valid graded grids

**Table 4:** Multi-report aggregate wear assessment results from five test reports.

Item	Value
Total analyzed units	4212
Mean composite wear score	0.711
Good	1881 (44.7%)
Slight wear	1475 (35.0%)
Moderate wear	318 (7.5%)
To be reviewed	538 (12.8%)

## Data Availability

The datasets generated and/or analyzed during the current study are available from the corresponding author upon reasonable request. Some raw road scene data cannot be publicly released because they contain geospatial information and roadway imagery that may be subject to privacy or infrastructure security considerations.

## Abbreviations

The following abbreviations are used in this manuscript:

GNSS	Global Navigation Satellite System
IMU	Inertial Measurement Unit
LiDAR	Light Detection and Ranging
LIO-SAM	Tightly Coupled LiDAR Inertial Odometry via Smoothing and Mapping
ROI	Region of Interest
ROS	Robot Operating System
TF	Transform
YOLO	You Only Look Once
